# Prostate cancer screening in Brazil: should it be done or not?

**DOI:** 10.1590/S1677-5538.IBJU.2015.0709

**Published:** 2016

**Authors:** Wilson F. S. Busato, Gilberto L. Almeida

**Affiliations:** 1 Departamento de Urologia, UNIVALI – Itajai, SC, Brasil; 2 Instituto Catarinense de Urologia, Itajaí, SC, Brasil

**Keywords:** Prostatic Neoplasms, Mass Screening, Brazil

## Abstract

The use of PSA in the screening, detection and prognosis of prostate cancer (PCa) has revolutionized the diagnosis and treatment of this disorder with an increase in detection rates and PCa organ-confined. Despite these benefits and ease of implementation, tracking PCa remains a matter of great controversy. We conducted a literature review and demographic and epidemiological data in Brazil feeling to assess the current state of screening and whether there is justification for population programs. the differences are valued between developed and underdeveloped countries as the incidence, mortality, screening and access to health. an analysis of the advantages and disadvantages of screening is made as well as a critical analysis of existing studies on screening and some recommendations on a rational screening.

## INTRODUCTION

The use of prostatic specific antigen (PSA) for screening, detection and prognosis of prostate cancer (PCa) had a big impact on diagnosis and treatment of this disease. It was observed an increase of detection rate, and particularly an tremendous increase of detection of organ-confined disease (1–5). From 1986 to 1993, the incidence of PCa increased from 86 to 179 cases/100.000 white men and from 124 to 250 in black men. The rate of metastasis at diagnosis dropped from 15 to 6.6 cases/100.000 (6). Although with clear benefits and easy to perform, PCa screening is very controversial. In the north hemisphere countries, where screening protocols were applied at populations at risk, it was observed an initial reduction of morbidity and mortality rates followed by a stagnation of these rates. This epidemiologic kinetics, although with low level of evidence, lead the American Urological Society to not recommend the use of vast screening of risk groups in North America (7).

The recommendation was based on a decision of the United States Preventive Services Task Force (USPSTF) (8) (grade D recommendation) mainly based on the contrary results of two major studies: European Randomized Study of Screening for Prostate Cancer (ERSPC) (1) and Prostate, Lung, Colorectal, and Ovary Cancer Screening Trial (PLCO) (9). That recommendation need to be worldwide validated. PCa screening rate (SPCa) is higher in comparison in USA and western Europe than in other countries, where it has been applied systematically (10). In under-developed countries, such as Brazil, there are no government SPCa rates and the numbers are opportunistic, according to information of population studies derived from isolated municipal campaigns (11). In 2008, it was estimated that there were 899.000 new cases of PCa documented in the World and 258.000 deaths. The estimate for 2030 is 1.7 million cases with 500.000 deaths (12).

Internationally, it is observed a wide range of incidence and mortality rates, mainly due to early diagnosis based on PSA screening. Variation of incidence among countries may be up to 24 times and of mortality of up to 10 times (12). It is important to correlate those data with other populations, in particular in less developed countries, with different ethnic background and economical status, in a different historical moment in terms of SPCa.

We performed a literature review of demographic and epidemiologic data from Brazil in order to evaluate the current status of SPCa trying to stablish the need or not of population screening programs. This review may provide data for government and medical societies decisions for screening programs for underdeveloped countries.

### Screening of prostate cancer in Brazil

Very little is known about SPCa in Brazil. Decisions are made mainly based on data collected in other countries with a wide geographic range. A world analysis of PCa incidence by 100.000 inhabitants, according to six groups of countries, showed that USA, Australia and Northern Europe had the highest incidence (83.2 to 173.7), followed by Brazil (45.3 to 83.1). However, these data invert when applied to mortality rates. USA are the fourth group (7.5 to 11.5), but Brazil, Australia and Northern Europe remain in the second place, with 15.3 to 22 deaths by 100.000 inhabitants (12). That means that in North America there is a high incidence of PCa but with low mortality (11). Recently, a Spanish study, where the incidence and mortality are similar to USA, showed no benefit of screening on mortality, justifying the theory that locals with low mortality do not benefit from screening (13). In Sweden and Denmark, with the same mortality rates than Brazil, screening lead to lowering of the rate more recently (2, 3).

It is known that under-notification is a serious problem in Brazil. Officially, the National Institute of Cancer estimated in 2014 that the Southern region of Brazil was more affected, with 91/100.00 inhabitants, followed by the Southeastern (88/100.000), Midwest (63/100.000), Northeastern (47/100.000) and Northern region (30/100.000) (14). Data of Sao Paulo Population Cancer Registry (2000-2005) showed a PCa rate of 337/100.00 in men with 60-64 years old and of 1.137/100.000 in men with 80-84 years (11). Other studies showed rates of 112/100.000 (Brasilia) and 99.3/100.000 (Goiania) (11, 15–19), higher than those published internationally. This wide variation may reflect the differences of health access in our country, low notification and possibly any specific population variation still unknown.

In Brazil, there is no active population screening such as those for breast and uterine cervix cancer. Some men are submitted to exams when spontaneously seek medical attention (16). In 2011, the National Health System of Brazil (SUS) paid 17 million gynecological consultations and only 2.6 million urological consultations (DATASUS). Male population from 45 to 75 years old, the target population for screening, was around 21 million in 2011 and SUS paid 3.9 million PSA exams (total and free). Since many of these exams referred to patients already diagnosed with PCa for follow-up purpose (around 1.7 million patients (17)), many men performed more than one exam/year without medical referral, a significant amount of patients performed the exam privately, and it is possible to infer that less than 15% of that population dose PSA. In Brazil, there are three major health systems: the public one, responsible for 76% of medical care; complementary, including medical insurance companies responsible for 23% of population; and the private system, responsible for 1% (Health Ministry, 2010).

A study involving 135 physicians with ≥51 years old from the Medicine School of the Federal University of Minas Gerais showed that 21% never dosed PSA (15). In Brazil, the reasons for refusal of PSA dosage and rectal exam include: age <70 years, less than 8 years of schooling, per capita income lower than 0.5 minimum wages (11). Among other countries, these rates are much higher. In the US, around 67% of men ≥60 years old had collected PSA in the last 12 months. In another American study based on the 2000 census, 62% of men ≥65 years old dosed PSA in the last year (11). At Tirol, after PSA became available in public health system, 86.6% of men performed the exam. In Japan, screening is similar to ours, 10% of men perform the test. However, we cannot state that there is or there was SPCa in Brazil.

Prostate cancer is usually more aggressive in lower ages and in blacks/browns, the majority of our population (Brazilian Institute of Geography and Statistics, IBGE 2010: 97 million), different from the USA population. Fortunately, we live in a country with high miscegenation. According to Darcy Ribeiro (20), there is an “in common multi-ethnic genetic background” and it is very difficult to distinguish men according to race. A recent research by the Catholic University of Brasilia showed that 45% of all Brazilians, black and whites, have 90% of African sub-saharan genes and that 86% present up to 10% of African sub-saharan genes, but European ancestry predominates in 80% of individuals (21).

It should also be pointed out some aspects of men from rural zones. Even main global statistics on cancer (Globocan) affirm that they only deal with only 30% of world population. In Brazil, rural population is 16%, or 30.4 million, and there are 15 million of men (IBGE-CENSUS 2010). Besides, men who live in small cities and those marginally to big cities, as well as those from slum dwells, have very little access to medical attention. The timetable of public health services, the decoration with female and childish motives of public campaigns and the need of a second attendance for PSA collection and a third one for physician analysis of the results also contribute for those low number of men screened. At the evaluation of AMS 2009 (Medical Assistance, 2009, IBGE), only 5.2% of public health services without hospitalization had clinical exams available (22).

We should also rethink the strategy to not screen men with less than 10 years of life expectancy. We should rethink life expectancy in Brazil. According to IBGE, life expectancy in Brazilian at birth is 74.6 years; Santa Catarina is the state with higher life expectancy, 76.8 years. In the nineties, when most papers were published about survival of PCa, life expectancy was 66 years, and men died due to other causes not related to low morbidity and mortality of prostatic tumor (DATASUS). Moreover, these are data related to life expectancy at birth. We have to consider that, as long as we survive diseases and accidents, we prolong our life expectancy. According to SUS Health National System table used to guide benefits, that takes into account life expectancy from a determined age, a person with 55 years old would have a life expectancy of more 25.5 years, a person with 65 years more 18 years and with 70 years of more 14.6 years (18). Therefore, it is a mistake to estimate life expectancy of a person over 65-year old, adding inherent risks to a period anterior to his/her time line.

Several aspects must be discussed when we analyze screening above 70 years, such as co-morbidities and choice of treatment, aside from life expectancy. It is important to have in mind the aggressiveness of PCa. In a recent Swedish prospective study showed how low risk tumors treated conservatively presented a considerable risk of progression and death after 15 to 29 years (20/1000 persons/year and 17.9/1000 persons/year) (23).

### Does PSA-based screening have benefits?

Although with low rate of mortality, PCA kills many men in the World. In developed countries, median survival from diagnosis in 5 years is 64%, while in under-developed countries is 41%. World mean is 58%, with 258.000 deaths/year (12). Which are the possible benefits?

### Mortality reduction

**Figure 1:**
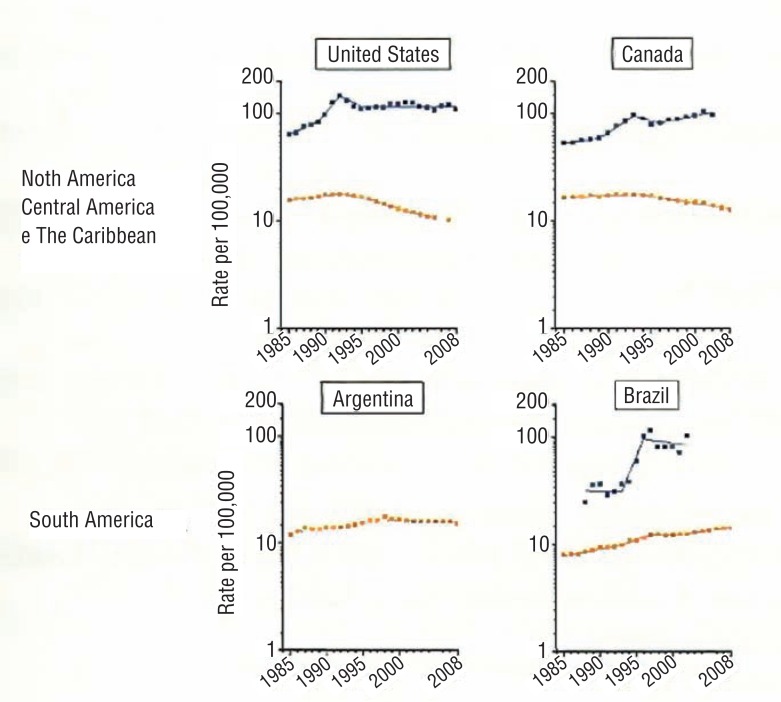
Incidence tendency and mortality of prostate cancer according to country (Adapted from center et al. (12))

According to the last cancer register in USA, mortality rate lowered 34% from 1990 to 2004 (16). Official data point that reduction after the beginning of PSA based screening and mainly due to PSA-based SPCa and early hormonal treatment derived from the higher number of diagnosis in less advanced stages (SEER - National Cancer Institute Statistics: http://seer.cancer.gov/csr/1975_2005/). Recently, tendency analysis of incidence and mortality of PCa in the last decades showed distinct figures among countries (Figure-1). While USA and Canada have implemented screening programs and the detection rates raised to a maximum point, reaching a diagnostic plateau, Brazil shows rising detection rates. Moreover, in those countries, the incidence increase of 7% per year of PCa reflects the real increase of incidence of the disease and not the extension of screening programs. And, more important, there was a reduction of mortality in the last decade. In relation to Brazil, mortality rates are still raising, showing that we are lagging behind in relation to screening. When we compare the curves, there is a difference of 20 years.

(blue: incidence; orange: mortality)

PLCO, with almost 77.700 participants, in their analysis of anticipation in 7 years of follow-up, showed a death incidence of 2/10.000 persons/year (50 deaths) among screened men versus 1.7 (44 deaths) in control group (9). They concluded that PCa related mortality in screened men is so low that screening is not justified (LE: 1B). A recent Cochrane analysis did not show any reduction on mortality in five randomized studies with a total of 341,342 participants, and stated that there is no reduction of mortality in up to 10 years of follow-up (24). However, these are small studies, with methodologic errors, with level of contamination and, mainly, follow-up inferior to 10 years. It is strange to realize that it was used studies with less than 10 years of follow-up to analyze a disease that does not kill before 10 years after diagnosis.

On the other hand, ERSPC, that included more than 162.000 men with 55 to 69 years old, found, in a follow-up of 11 years, a reduction of mortality of 21%, or 29% after adjustment. But the reduction was significant only for men between 65 and 69 years old. They concluded indicating a reduction of 38% of relative risk (RR) in a follow-up of 11 years, but with no reduction for men ≥70 years old (1). An update of the data from Goteborg section with 14 years of follow-up showed a reduction of RR of 50% of mortality in the screened group and 41% reduction of metastasis (2). However, this reduction was accompanied by an important increase of over-treatment. At the initial report of ERSPC, in order to prevent 1 death, they had to screen 1410 men and to treat 48. In the last update, with 11 years of follow-up, it was necessary respectively 1055 and 37. In the Goteborg study of 14 years, it was necessary to screen 293 and to treat only 12 patients in order to avoid one death.

### Reduction of cases with metastatic or more advanced disease

Sensitivity and specificity of PSA increase for high grade PCA, showing that it is a better marker for aggressive tumors than for low grade tumors. With screening, there is a higher number of localized disease, lowering the number of metastatic and high grade patients, in the order of 31% (25). Consequently, there is a reduction of pathological fractures, bone pain and the need of treatment, with positive impact on quality of life of those men and families. Prior to PSA era, only 27% of PCa cases diagnosed were localized. In the present, 97 to 98% of all PCas diagnosed through screening are localized (1, 9, 26). Before PSA, 75% of patients died due to cancer or it contributed to death. Mean survival time between diagnosis and death was 41 months (26).

The risk of metastatic PSA reduced 30 and 49% among men screened at ERSPC and Goteborg, respectively. In the last, 2.6% of screened and 10.6% of patients of control group had diagnosis of metastatic disease (2). The absolute risk reduction is of 3.1 cases for every 100 patients screened. Etzioni et al. (27) tried to stablish a reduction of metastatic cases after the introduction of PSA based screening program in a simulated model. With screening, the incidence of metastasis reduced from 77 cases/100.000 men in 1990 to 37 cases in 2000, corresponding to a reduction of 80% of advanced disease (27).

Many studies have shown a reduction of biochemical recurrence following radical prostatectomy in men diagnosed through screening (25) reducing the need of hormone therapy, radiotherapy and chemotherapy and their risks and complications as well as costs of theses auxiliary treatments.

**Figure 2:**
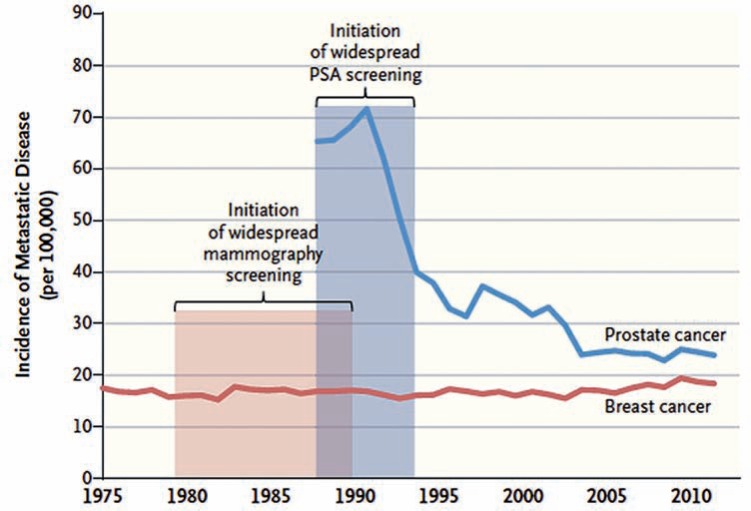
incidence of metastatic cancer at diagnosis, USA, 1975-2012.

Recently, one study about evolution of metastasis in prostate and breast cancer at diagnosis showed that screening reduced advanced cases of PCa but not of breast cancer. Unlike mammogram, PSA is one of the most efficient markers for high risk diseases, and the reduction of more advanced cases was obtained by screening, and not from reduction of risk factors (Figure-2) (28).

Breast cancer data (SEER) of women ≥40 years old, and prostate cancer (American Joint Committee Stage IV) of men ≥40 years of age (28).

### Determination of risk of prostate cancer throughout life

Three key questions are empirical and still not answered: 1) In which age should we start early detection of PCa? 2) In which interval should we perform PSA and rectal exam? And 3) Until which age should we screen for PCa? There are evidences that determination of PSA at 40-45 years is related to future risk of PCa (29, 30). EUA suggests that PSA dosage may be used to define posterior periodicity (LE: B) (31). PSA >1.0ng/mL at 45 years old is associated to a significantly higher risk of death related to PCa, as well as advanced or metastatic disease diagnosis even after 25 years of that dosage (21).

Medium level of PSA of healthy men (Table-1) varies according to age and several studies have shown that a higher PSA of a specific population according to age may be a better indicator for future development of PCa than other clinical risk factors, such as race, familiar history and suspect rectal exam (1, 4, 30, 32).

**Table 1:** PSA mean level in healthy men (29).

Age	PSA (ng/mL)
30 - 49	0.6 a 0.78
50 - 59	0.7 a 1.23
60 - 64	1.2
65 - 69	1.43

Västerbotten Intervention Project (33) followed 540 men and showed that among those with basal PSA <0.1ng/mL only 3.9% developed PCA and 1.2% with high risk tumor. Men with PSA from 1 to 2ng/mL have an odds ratio (OR) for PCa of 9.1%, from 2 to 3ng/mL of 23.3 and from 3-4ng/mL of 43.9%. Although that study did not stablish a cut-off level for prediction of PCa, it was able to determine that initial value <1ng/mL was associated with a very low chance to develop clinical tumor in the future. However, data obtained by the Malmo Preventive Project (30) indicate that screening should be applied in men even with basal PSA below medium, since 28% of metastatic PCa showed levels below medium for up to 27 years.

Even for men over 60 years old, PSA value can predict future risk of PCa, development of metastasis and death at 85 years old. Men in that age group with PSA ≥2ng/mL has 26 more times risk of death due to PCa than those with <2ng/mL (34, 35).

### Frequency of screening

Basal PSA, obtained from 45 to 59 years of age, can help urologist/oncologist to plan interval of PSA and rectal digital exams. An evaluation of the Rotterdam section of ERSPC indicates evaluation every 2 to 4 years for men with PSA >1.0ng/mL and of up to 8 years for values <1ng/mL. That study followed 1703 men with 55 to 65 years of age during 8 years with PSA ≤1.0ng/mL and diagnosed only 8 cases of PCa (0.47%) (36). The interval varied in the ERSPC study, and most centers used an interval of 4 years, and the Goteborg arm, 2 years. The interval of 2 years resulted in a reduction of death by PCa RR of up to 44% and of 43% for advanced disease. However, it increased the number of diagnosis of PCa and the incidence of low risk cancer, elevating overtreatment. Although there is still no clear definition of the precise interval the urologists must individualize it based on basal levels of PSA and on co-morbidities and life expectancy of their clients (1).

### Does PSA-based screening have risks?

#### Diagnosis of low important disease (overdiagnosis)

Some fear that PSA based SPCa could lead to excessive diagnosis, and consequently, overtreatment, with a sequence of unfavorable facts and events and lowering quality of life (5, 37). This is one of the reasons that made USPSTF be unfavorable to SPCa (4). In the last decades, a few steps were introduced in diagnosis and treatment of PCa. Only PCa locally advanced were operated at EDR. After the introduction of PSA, all tumors are treated and diagnosed without distinction, with overdiagnosis and overtreatment. After a sequence of natural learning, many PCa diagnosed by PSA may and must be submitted to active surveillance.

**Figure 3:**
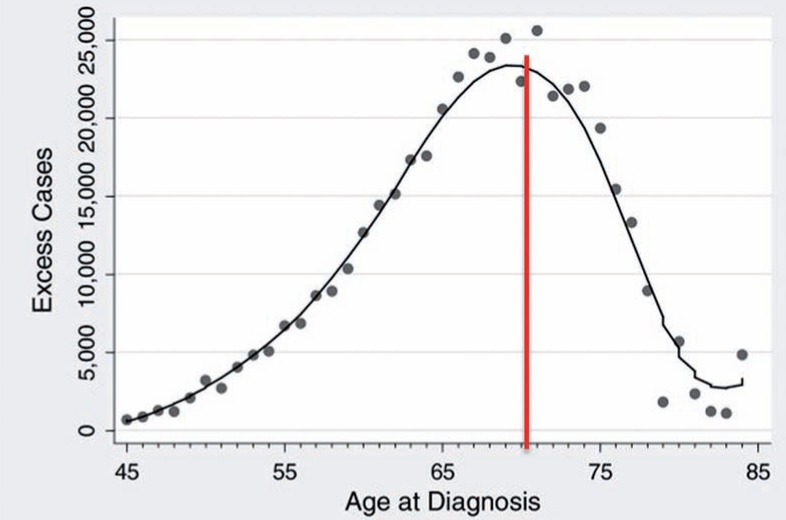
Number of cases with excessive diagnosis of prostate cancer according to age, from 1987-1995 (47).

Frequently, the studies report overdiagnosis when analyzing PSA results as a whole, regardless age (38). Almost half unnecessary diagnosis by PSA are made in men older than 70 years, an age group with very few indications of screening (Figure-3). In a simpler way, only controlling that practice would reduce in half overtreatment. Another measure would be to restrict screening in men >60 years old to those with PSA >1ng/mL, informing those with lower age that an eventual PCa diagnosis would rarely cause death.

Recently, data from the Prostate Cancer Intervention Trial (PIVOT) (4) were published. They randomized 731 men to radical prostatectomy (RP) and observation. Some conclusions regarding screening were made. After 10 years, mortality among participants with PSA ≤10ng/mL was 46% with RP vs. 44% for observation (RR 1.06; 95% CI, 0.87-1.29), but for men with basal PSA >0ng/mL it was 48% for RP vs. 62% for observation (RR, 0.79; 95% CI, 0.63-0.99). These data suggest that it could be adopted an increase in the traditional threshold for biopsy indication without prejudice to treatment, avoiding diagnosis with low PSA of a non-clinical significant disease (39).

### Adverse effects of screening and treatment

Recent studies have shown a rate of moderate/severe adverse effects in men submitted to biopsy (TURPB) relatively low, such as hemospermia (27%), pain (75), hematuria (6%), fever (6%), hematoquezia (2%), and risk of hospitalization of 0.5-1.3% (40, 41). However, population studies show that this hospitalization rate can reach 7% (42). Per-operatory mortality of PC is around 0.5% and absolute risk of urinary incontinence is 11%, and erectile dysfunction 43%. In the long term follow-up, surgical and radiotherapy complications are similar (39).

### Difficulties of the background studies

#### Follow-up time

As referred above, grade D recommendation of AUA and of United States Preventive Services Task Force (USPSTF) (43) was based mainly in two studies, with conflicting results (Table-2). ERSPC study had a great number of participants with an adequate follow-up and PLCO had half participants and partial publication of data (44, 45). Both studies had short duration. It is believed that any alteration of the natural slow history of PCa should occur only after 11.5 years (46). One of the long arms of ERSPC (Goteborg Trial) with 14 years of follow-up showed reduction of 44% of mortality among screened men. The great paradox is that AUA's SPCa guidelines for more than a decade indicate that we must not screen men with life expectancy ≤10 years, but when it evaluated SPCa benefits they based on studies with shorter follow-up. We must remember that those studies were planned 20 years ago, when the knowledge of natural history of PCa was a fraction of what is known nowadays.

**Table 2:** Comparison of two major studies of prostate cancer screening.

Item	ERSPC (Schroder et al.) (1)	PLCO (Andriole et al. (9)
Sample	182,000 men 50 to 74 years old	76,693 men
Local	European countries	10 North-American center
Biopsy	PSA > 3ng/mL	PSA > 4ng/mL
Follow-up	9 years	7 years, with 10 years with only 67% of data
% biopsy	85.8%	40.2% (7 years) and 30% (10 years)
Stage	Most PCas in both arms were stage I	Most PCas in both arms were stage II
Gleason	Most in both arms were Gleason 2 to 6 (at biopsy)	Most in both arms were Gleason 5 and 6 (at biopsy)
Mortality	Reduction of 20% in the screened group, 31% in the arm really screened. Goteborg arm with 14 years and 44%	Low mortality in both arms, without difference

(Adapted from Bailey et al. (44) and Schoder et al. (45)).

Since most deaths occur in men over 70 years old (47) and both studies included men >50 years, ideal follow-up time to determine reduction of mortality should be longer than 14 years, since many participants did not reach the group age when death occurs.

### Tumor data

PLCO did not find advantages with screening and involved a smaller number of patients with more advanced disease and with Gleason score lower than ERSPC (1, 9). With a larger number of PCa with low or intermediate risk, the tendency is low mortality. It is known that for these groups screening is not essential. This “aggressiveness shift” may be revealed by the published low mortality (<0.3%). There were 174 tumor-specific deaths at PLCO. That fact was observed since almost half participants had at least two previous PSA exams, and those with PCa diagnosis were not included in neither study arms, being pre-selected.

### Power of the study

We must remember that at analysis of strength of PLCO, 37.000 men should be initially evaluated in each arm during 10 years. However, only 67% of mortality data were available in that period. Besides, changes after the beginning of the protocol in 1995, such as reduction of access age to 55 years, resulted in reduction to accuracy and new calculations in order to maintain accuracy, with the need of follow-up of more 13 years from the last modification, that occurred in 2011. Only these two aspects, according to statistics, reduce the power of the study in 50% (48).

### Study contamination

One of the methodological bias of PLPO was that almost half candidates admitted in the group control of the study had already performed occasional PSA dosages. This situation is common in regions where screening has already been implemented. This previous evaluation works as a “pre-selection” and a selection bias, since many PCas had already been identified, and therefore, were left aside from the study. One proof is the presence of lower number of patients with advanced PCa and/or high Gleason score (≥7) than other publications (4).

It is accepted a maximum of 20% of contamination, but at PLCO almost half participants had performed a previous PSA exam. Therefore, under an epidemiologic point of view, we can affirm that PLCO compares two kinds of screening: more and less intense (9).

### What will happen if we stop screening?

If we suspend screening (Brazil never had screening) indiscriminately we will eliminate the risks but also the benefits (49). One of the reasons for the reduction of morbi-mortality of PCa of PSA-based screening was downstaging (36) at diagnosis. If we stop screening favorable effects will no longer persist (5). However, since 93% of deaths due to PCa occur in men >70 years old (50), period in in which a more number of high grade of PCa are diagnosed, the suspension of screening in men <65 years will increase morbi-mortality in that age group in a few years (51).

Bergdahl et al. (51) followed 13.423 men: half of them stopped screening at 69 years of age and half (control group) never were screened. In the first 9 years of the last PSA all risk groups of PSA were more frequently diagnosed among those never screened, but after 9 years, the rates were the same, except for groups with low risk. The reduction of advanced and high risk PCa observed in the screened arm during the screening period lasted for 9 years. After that period, mortality rate of those screened reached that of the group never screened (51). Therefore, after 9 years, the welcome displacement of stage to less advanced disease and with lower risk was lost. Also interesting, the authors noted a gradual drop of the protective effect of screening, starting after 4 years of the last PSA. The authors informed that, as a consequence of suspending screening at 69 years of age, men ≥78 years will have the same risk of advanced of high risk disease than those not screened.

### Rational use of PSA

If the specialists do not agree if we should or not perform screening, we cannot expect patients to decide for that measure with less information. AUA guidelines suggest that we should screen patients who understand and accept the risks. However, this approach could result in different health care management, since well-educated and informed men would ask for screening and would be effective screened (37). And certainly that recommendation would not be applied to the majority of the Brazilian population, in view of the disparity of socio-economic status of our population than that of USA.

More recent data indicate that the real discussion is not if we should perform PSA based screening of PCa but how to do it rationally. Most authors agree that annual screening of all men ≥50 years old is not justified (26, 44, 52–54). Screening programs must focus on the decision capacity of PCa experts (urologists and oncologist) and not on basic health programs. Table-3 shows some guidelines that help perform a more smart screening.

**Table 3:** General measures that may help define guidelines for prostate cancer screening.

PSA dosed at 45-50 years old may identify risk groups and indicate screening interval Reduce intensity of screening > 60 years old in men with PSA<2.0 ng/mL There is little benefit to screen men>70 years old, with PSA≤3.0 ng/mL and with two or more co-morbidities Smart screening in men 50-69 years old Screening intervals from 2/2 years up to 7/7 years, individualized Attention to familial prostate cancer, defined as two first-degree relatives or on first-degree relative and at least two of second-degree Attention to screening in morbid obeses and blacks with good health Population screening for all men is not justified Urologist/oncologist must individualize need and method of screening Consider active surveillance/observation for patients with low risk PCa
